# Natural Killer Cells as Allogeneic Effectors in Adoptive Cancer Immunotherapy

**DOI:** 10.3390/cancers11060769

**Published:** 2019-06-03

**Authors:** Kyle B. Lupo, Sandro Matosevic

**Affiliations:** 1 Department of Industrial and Physical Pharmacy, Purdue University, West Lafayette, IN 47907, USA; klupoo@purdue.edu; 2 Purdue Center for Cancer Research, West Lafayette, IN 47907, USA

**Keywords:** natural killer cells, graft-versus-host disease, HLA mismatch, allogeneic immunotherapy

## Abstract

Natural killer (NK) cells are attractive within adoptive transfer settings in cancer immunotherapy due to their potential for allogeneic use; their alloreactivity is enhanced under conditions of killer immunoglobulin-like receptor (KIR) mismatch with human leukocyte antigen (HLA) ligands on cancer cells. In addition to this, NK cells are platforms for genetic modification, and proliferate in vivo for a shorter time relative to T cells, limiting off-target activation. Current clinical studies have demonstrated the safety and efficacy of allogeneic NK cell adoptive transfer therapies as a means for treatment of hematologic malignancies and, to a lesser extent, solid tumors. However, challenges associated with sourcing allogeneic NK cells have given rise to controversy over the contribution of NK cells to graft-versus-host disease (GvHD). Specifically, blood-derived NK cell infusions contain contaminating T cells, whose activation with NK-stimulating cytokines has been known to lead to heightened release of proinflammatory cytokines and trigger the onset of GvHD in vivo. NK cells sourced from cell lines and stem cells lack contaminating T cells, but can also lack many phenotypic characteristics of mature NK cells. Here, we discuss the available published evidence for the varying roles of NK cells in GvHD and, more broadly, their use in allogeneic adoptive transfer settings to treat various cancers.

## 1. Introduction

In recent years, results from clinical studies have demonstrated safety and efficacy of allogeneic infusions of natural killer (NK) cells for immunotherapy of hematological malignancies and solid tumors [1]. NK cells are innate immune effectors whose anti-tumor activity is regulated by a complex interplay of a large variety of inhibitory and activating receptors [2]. These inhibitory receptors, which include killer immunoglobulin-like receptors (KIRs) and CD94/NKG2A, are able to recognize major histocompatibility complex (MHC) class I molecules determined by human leukocyte antigen (HLA) HLA-A, HLA-B, HLA-C or HLA-E allotypes [3]. Encoded by genes on different chromosomes, this allows for donor and recipient mismatching between KIRs and their ligands, allowing control of NK cell activation in immune responses and their alloreactivity as allogeneic effectors.

The use of NK cells in allogeneic immunotherapy benefits from these cells’ short persistence, their assumed role in the depletion of alloreactive T cells, and their alloreactivity induced by the mismatch between KIR receptors and their ligands on target cells [4]. In addition, alloreactive NK cells do not express inhibitory receptors specific for HLA-class I alleles on target cells [5,6]. Allogeneic NK cells have shown clinical benefits against a number of cancers, particularly against acute myeloid leukemia (AML), after both hematopoietic stem cell transplantation (HSCT) and allogeneic infusions of isolated NK cells [7]. Allogeneic NK cells from healthy donors have the advantage of being fully functional. In allogeneic HSCT settings, donor T cells are responsible for contributing to graft-versus-host disease (GvHD) and graft-versus-tumor (GvT) responses [8]. NK cells, on the other hand, are thought to mediate GvT effects in the presence or absence of donor T cells with a limited induction of GvHD [9] and have been used in settings of T cell-depleted or T cell replete HSCT. Sources of allogeneic NK cells include peripheral blood, cord blood, and bone marrow [10].

Despite the immune-protective effect that NK cells appear to exert following adoptive transfer in both transplant and non-transplant settings, their roles within GvHD and anti-tumor immune responses are not fully clear. Traditionally, the GvHD suppressive role of NK cells has been thought to be exerted by their cytolysis of T and dendritic cells [11,12,13]. However, conflicting reports have questioned their exact contributions to GvHD. More specifically, reports have shown that cytokine stimulation required for NK cell expansion and activation can mediate GvHD through activation of T cells and NK cells’ secretion of pro-inflammatory cytokines [14,15,16], thereby limiting safe, efficacious use of peripheral and cord blood-derived NK cells in adoptive transfer settings.

Other NK cell sources, such as induced-pluripotent and human embryonic stem cells (iPSCs and hESCs) and NK cell lines offer the benefit as a source of NK cells, free of contaminating T and B cells, mitigating any alloreactive effects and GvHD associated with blood-derived NK cells [1]. However, challenges in procurement and sourcing of these cells currently limit their widespread use as clinical NK cell therapies. Nonetheless, NK cell lines in particular have proven promising for use in adoptive transfer setting, with a number of currently ongoing clinical trials.

## 2. Immunobiology of Target Recognition by Natural Killer Cells

### 2.1. Target Recognition and NK Cell Activation

**Figure 1:**
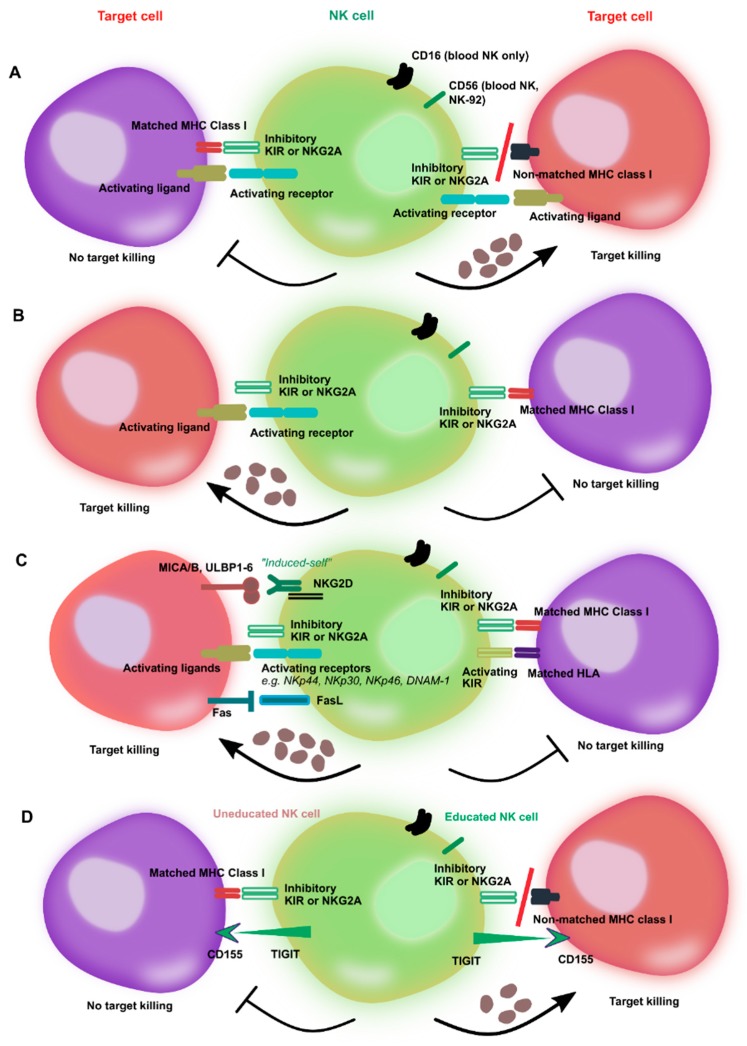
Activation of natural killer (NK) cells by inhibitory and activating receptors and modes of killing of target cells. (**A**) Recognition by inhibitory killer immunoglobulin-like receptors (KIRs) of major histocompatibility complex (MHC) class I molecules on cancer cells inhibits NK cell killing due to “missing self” recognition despite the presence of activating receptors. The balance between inhibitory and activating signals does not induce NK cell activation. However, NK cells are triggered to kill their targets when no matching KIR ligand is present, which shifts the balance toward NK cell activation. (**B**) The lack of MHC molecules prevents inhibition of NK cytotoxicity and promotes NK-induced killing of targets by activating receptor engagement. However, MHC ligand mismatch is not by itself sufficient to trigger NK cell killing in absence of signals from activating receptors. (**C**) Many cancer cells dowregulate the expression of MHC ligands. Even with the lack of MHC ligand expression, the balance of activating receptors in favor of killing signals can trigger NK-mediated lysis of cancer targets. Activation is further promoted by the recognition of stress ligands on cancer cells, as well as “induced self” ligands, such as MICA/B and ULPB1-6 for NKG2D. Conversely, a balance between activating (e.g., KIR2DS) and inhibitory KIRs promotes NK tolerance and results in no killing. (**D**) The process of NK cell education imparts NK cells with functional maturation and self-tolerance. The example of the TIGIT/CD155 interaction is shown: Only educated NK cells can mediate killing by engagement of CD155/TIGIT ligation. The hyporeactivity of uneducated NK cells, similarly to recognition of MHC class I by inhibitory KIRs, ensures NK self-tolerance.

NK cells mediate their anti-tumor immunity based on the net balance of inhibitory and activating receptors (Figure 1) [17,18]. Target cell killing mediated by NK cells does not occur by default in the absence of inhibitory receptor engagement, but requires the presence of activating receptors to stimulate cytotoxicity. Traditional inhibitory receptors involved in NK cell responses belong to either KIRs or CD94/NKG2A families. KIRs include KIR2DL1, KR2DL2, KIR2DL3 as well as KIR3DL2 and KIR3DL3. They bind to HLA-C (KIR2DL1, KR2DL2, KIR2DL3) or HLA-A and HLA-B (KIR2DL1, KIR3DL2 and KIR3DL3) on target cells, while NKG2A—expressed both on mouse and human NK cells—recognizes the non-classical ligand HLA-E. The extent of such NK cytotoxicity is inversely proportional to the level of expression of HLA ligands on target cells [19]. However, MHC class I ligands are not the only inhibitory ligands detected by NK cells, as other inhibitory receptors that recognize MHC-independent self molecules on cancerous and distressed cells have been identified, including 2B4, CEACAM1, KLRG1, and LAIR1 [20]. In addition to these receptors, NK cells express a variety of other MHC class I-independent surface molecules which have been recognized as checkpoints with roles in further directing NK cell cytotoxicity, such as T cell immunoreceptor with Ig and ITIM domains (TIGIT) and programmed death-1 (PD-1) [21].

Upon recognition of a target cell, NK cells form an immunological synapse guided by the interaction between their many receptors and cognate ligands on target cells [22]. Synapse formation following ligand engagement is accompanied by rearrangement of the actin cytoskeleton, and triggers phosphorylation of immunoreceptor tyrosine-based inhibition motifs (ITIMs) in the cytoplasmic tail of inhibitory receptors by Src family tyrosine kinases [23]. It bears mentioning that clustering of KIRs and NKG2A is independent of actin polymerization and ATP [24]. This results in the recruitment of Src homology 2 domain-containing protein tyrosine phosphatase-1 (SHP-1) and SHP-2 [25], which drive and are required for inhibition of NK cell function [26]. The presence of inhibitory receptors on NK cells and the corresponding ligands on target cells act as a sort of safeguard mechanism to prevent unfettered cytotoxicity of NK cells in vivo. Expression of stress ligands on target cells can further shift the balance toward NK cell activation, ultimately resulting in lysis of those cells via the NK cell-mediated release of cytotoxic granules. NK cell inhibitory receptor accumulation at immunological NK-target cell synapses is rapid [27].

### 2.2. NK Cell Activation by Receptors, Ligands, and Co-Receptors

“Missing self” is not by itself sufficient to induce killing of targets by NK cells. It is the overall balance between inhibitory receptors including KIRs and NKG2A with the level of expression of activating KIRs (for example, KIR2DS or KIR3DS) or NK receptors such as NKG2D, NKp44, NKp46, NKp30, NKp65, NKp80, CRACC or LFA-2 as well as DNAX accessory molecule 1 (DNAM1) that determines the extent of NK-mediated cytotoxicity [28]. NKG2D is capable of recognizing stress-induced self-ligands (“induced self”), which are not present or expressed at low levels on healthy cells, but are upregulated on distressed or cancerous cells [29], such as MHC class I polypeptide-related sequence A/B (MICA/MICB) and UL16 binding protein 1-6 (ULBP1-6) in humans, and has been shown to mediate in cancer killing. Additionally, the Fc receptor CD16 expressed on NK cells functions to mediate cytolytic activity through recognition of antibody-coated cancerous cells [30]. The NK cell natural cytotoxicity receptors, NKp30, NKp46, and NKp44, have also been implicated in playing a key role in NK cell effector function, but corresponding ligands have yet to be clearly identified [31]. DNAM1 interacts with the adhesion receptor leukocyte function-associated antigen-1 (LFA-1), which is expressed on healthy cells, but largely inhibited through MHC class-I regulation, and upregulated on cancerous and distressed cells, and, therefore, mediates NK cell adhesion with target cells [31]. The tolerance to self and the activation of NK cells in response to pathogens is regulated by the process of education. NK cell education determines how NK cells respond to infected, stressed or pathogenic cells [32]. Since the process of NK cell education varies among individuals, responses to pathogens vary widely, resulting in divergent responses to disease and treatment. Donor selection based on education status enables modulation of NK alloreactivity. Recent evidence has shown that education via expression of inhibitory KIRs drives the lysosomal rearrangement of lytic granules which in turn drive powerful NK effector responses [33].

### 2.3. Challenges with Solid Tumors

Despite the highly-controlled activation of NK cells in response to infected targets, NK cell activation in solid tumors presents particular challenges not present with hematological malignancies. Solid tumors are very heterogeneous, characterized by different gene profiles and mutations, which result in differing metastatic and proliferative potentials [34]. Solid tumor microenvironment-specific immunomodulators such as hypoxia, adenosine, lactate, and transforming growth factor-β (TGF-β) rearrange the repertoire of NK receptors and are able to induce down-modulation of activating NK receptors, further compromising NK cell-mediated cytotoxicity [35,36]. Additional complicating factors, such as poor intra-tumoral infiltration of NK cells and severe metabolic reprogramming that occurs in response to rapid glycolytic fueling by cancer cells leads to a dysregulated NK cell immune response and poor efficiency of adoptive immunotherapies. Though NK cell-mediated anti-tumor responses have been described for a number of cancers [37], with some studies showing correlation between intra-tumoral presence of infiltrating NK cells and better disease prognosis [38], insights into many cancers are either not known or are inconclusive. Exact mechanisms and manipulation strategies to durably and reproducibly enhance NK cell function in vivo are not known.

Strategies aimed at improving adoptive transfer of NK cells to solid tumors have included combination treatments with checkpoint inhibitors [39], genetic engineering to improve the targeting of NK cells via the expression of synthetic genes such as chimeric antigen receptors [40] or the expression of chemokines which can improve NK cell migration and trafficking into tumors [41], and combination treatments with cytokines, immunomodulatory drugs, antibodies, and oncolytic viruses [42,43]. With all that said, alloreactivity of NK cells in solid tumors remains a topic of high interest [44].

## 3. Allogeneic NK Cell Immunotherapy

### 3.1. Allogeneic NK Cells in Hematopoietic Stem Cell Transplantation

Autologous NK cell activity is inhibited in cancer patients largely due to KIR ligand (KIR-L) match [45]. On the other hand, KIR-L/HLA-C mismatch in hematopoietic transplants was shown to mediate a more powerful anti-tumor response by triggering NK cell alloreactivity, augmenting HSCT, and potentially limiting GvHD [46,47,48]. NK cells are the first lymphocytic population to be reconstituted following allogeneic HSCT [49]. Peripheral blood is the most common source of cells for HSCT. It is commonly mobilized using granulocyte-macrophage colony-stimulating factor (GM-CSF) to produce hematopoietic stem cells [50]. Other than peripheral blood, cord blood or bone marrow are also used as sources of cells for HSCT. Typical immune suppression of recipients prior to HSCT involves a nonmyeloablative lymphodepleting conditioning regimen with cyclophosphamide and fludarabine [51]. After HSCT, reconstitution of NK cells occurs within one month irrespective of whether the cells for HSCT have been sourced from the bone marrow, umbilical cord blood or GM-CSF-mobilized peripheral blood, and regardless of donor type or patient age. Four selection approaches have been described for selection of HSCT (or NK cell) donors based on donor and recipient KIR and/or KIR-ligand genotypes: ligand–ligand mismatch, receptor–receptor mismatch, receptor–ligand mismatch, and by haplotype B score [52]. Following HSCT, the CD56^bright^ subset was reported to be the first to appear post-transplantation—this was particularly true for patients lacking GvHD―with CD56^int^ NK cells (which represent an intermediate state between CD65^bright^ and CD56^dim^) appearing three months post-HSCT, followed by CD56^dim^ cells. Unlike the former, expression marker profiles of CD56^dim^ cells were shown to differ in expression of KIRs, CD62L, NKG2A, and CD57 compared to those of CD56^bright^ and CD56^int^ cells [53]. A number of clinical trials are currently ongoing evaluating such an allogeneic NK-based immunotherapeutic modality (Table 1).

The contributions of NK cells to GvT effects post HSCT have been described. HLA-haploidentical HSCT (haplo-HSCT) for high-risk acute leukemia patients transplanted from NK-alloreactive donors has shown robust clinical outcomes [54]. NK cells reconstituted after haplo-HSCT into patients with acute myeloid, chronic myeloid or chronic lymphoblastic leukemia displayed the same KIR repertoire as the donor, developed tolerance for the host a few months after the transplant, and engaged in alloreactive target killing in the absence of GvHD [47]. Separate studies have demonstrated alloreactivity of NK cells in other settings as well: in the context of both HLA-matched [55] and HLA-mismatched [46] hematopoietic transplants. KIR genes, it should be noted, are likely to remain mismatched even for fully-matched HLA transplants [56].

Safety and tolerability of alloreactive NK cells post-HSCT have been the subject of much work. Though NK cells reconstitute rapidly after HSCT, they show delayed functional maturation for at least six months, which is reflected in lower production of pro-inflammatory cytokines interferon-γ (IFN-γ) and tumor necrosis factor-α (TNF-α) [57]. Generally, engraftment of NK cells from donor peripheral blood progenitors following non-myeloablative conditioning has been shown to correlate with a lower risk of relapse, independent of donor match or disease [58]. To improve safety and prevent the onset of GvHD in HSCT, recent strategies have included administration of post-transplantation cyclophosphamide [59]. Pende et al. [6] showed that alloreactive NK cells post-haplo-HSCT in pediatric leukemia patients persisted for years, and their anti-leukemia effect was dependent on activating receptor KIR2DS1 alongside alloreactivity induced by inhibitory receptors. While other studies have also indicated that NK cell infusions post-HSCT had no effect on tumor relapse or graft failure and were well-tolerated [60,61,62,63,64], more recent evidence hints at a potentially more complex relationship between KIR-mismatch and safety profile. Hosokai et al. [65] described the incidence of grade IV GvHD in A/A haplotype patients transplanted from HLA-mismatched donors with KIR haplotype B/x. In general, however, many studies have reported that better treatment outcomes correlated to higher NK cell numbers [65,66,67,68,69]. Donor selection was cited as a factor in a recent Phase II study which reported good safety and no GvHD, but no significant efficacy, in patients with relapse of persistent myeloid malignancies treated with haploidentical NK cells [70]. Though most of these studies were carried out in the context of hematologic malignancies, successes with solid tumors remain limited [71].

### 3.2. Allogeneic NK Cell Infusions

Adoptive transfer of autologous NK cells has been carried out to treat a number of diseases, including various solid tumors clinically (Table 2). However, autologous infusions of NK cells have failed to show a sustained anti-tumor response, despite demonstrated safety [72,73,74,75]. Combination with chemotherapy has, nonetheless, shown somewhat more promising results in patients with colon carcinoma [76]. Similarly, a number of clinical studies have demonstrated the safety of infused allogeneic NK cells to treat both hematologic malignancies and solid tumors [1]. These studies utilize allogeneic NK cell products that include in vitro cytokine and feeder cell expanded NK cells, non-expanded cytokine-activated NK cells, and cytokine-induced memory-like NK cells, which are generated after a pre-activation period with combinations of the cytokines interleukin (IL)-12, IL-15, and IL-18 and have the ability to functionally persist long-term in vivo [77]. 

It has been widely reported that NK cell functionality is heavily influenced by their pre-activation regimen prior to adoptive transfer [78]. Commonly, combinations of one or more cytokines including IL-2, IL-12, IL-15, IL-18, and IL-21 [79,80] are used to activate NK cells, with or without anti-CD3 stimulation [81]. Among them, IL-2 [82] has been associated with increased NKG2D expression, while IL-15 [83] is a powerful driver of NK cell differentiation and proliferation and, when used in combination with IL-2, has been shown to improve NK cell viability ex vivo. IL-18 was shown to stimulate IFN-γ production by NK cells and provide co-stimulatory activation [84], while IL-21 [85] was able to enhance maturation of NK cells without promoting proliferation. Feeder cells employed for expansion and activation of peripheral blood NK cells ex vivo have included irradiated autologous peripheral blood mononuclear cells (PBMCs) [83] and K562 cells expressing membrane bound 4-1BB ligand [86] and/or IL-15 or IL-21 [87,88]. Optimal protocols, and the interplay between specific cytokine programs with anti-CD3 stimulation, is, however, not fully clear.

Ex vivo-expanded, allogeneic NK cells have to be cultured until sufficient numbers of cells for infusion have been obtained. The first demonstration of the feasibility and safety of adoptively-transferred NK cells into patients was made by Miller et al. [89]. Their trials showed that adoptively transferred human NK cells derived from haploidentical related donors could persist in vivo and mediate anti-tumor effects in acute myeloid leukemia patients when KIR ligand-mismatched donors with recipient tumor MHC were used. Since then, additional studies with mismatched adoptively transferred NK cells were shown effective in high-risk elderly patients, adults, and children with acute myeloid leukemia [90,91,92] and non-small cell lung cancer [93], while limited in vivo persistence was described for haploidentical NK cell infusion with and without total body irradiation in patients with ovarian and breast cancer [94]. Conditioning regimens in these trials have included high-intensity and high-dose cyclophosphamide and fludarabine administration. Yang et al. [95] reported results of a recent Phase I trial evaluating the safety and efficacy of unrelated mismatched NK cells adoptively transferred into patients with either lymphoma or solid tumors. They found the maximum tolerated dose of NK cells to be 3 × 10^7^ cells/kg (triple infusion), while these cells persisted in the peripheral blood of recipients for up to four days. Despite the cells’ relatively short lifespan in vivo, the study demonstrated that these cells were both safe and somewhat efficacious, with 8 out of 18 treated patients showing stable disease.

Evidence that cytokine activation regimens are likely tumor- and conditioning protocol-specific came early. Bachanova et al. [96] reported that IL-2-preactivated allogeneic NK cells could lead to remission if refractory lymphoma patients were infused with IL-2 every two days for 2 weeks. Despite the initial positive response, however, remission was not durable. A Phase I/II trial of patients with lymphoma and breast cancer treated with ex vivo IL-2-activated autologous NK cells similarly failed to show disease response. On the other hand, 75% of patients with lung cancer treated with allogeneic NK-92 cells at a dose of 10^10^ cells/m^2^ showed an anti-tumor response [97]. In a separate study, adoptive transfer of haploidentical NK cells, pre-activated overnight with IL-2, showed a partial anti-tumor response in 20% of ovarian and breast cancer patients, and stable disease in 60% of patients [94]. Some responses were also seen from a recently-reported Phase I trial of patients with advanced solid tumors treated with ex vivo-expanded, IL-2 pre-activated NK cells from random unrelated donors. Additionally, 37% of patients treated with triple injections of 3 × 10^7^ cells/kg showed stable disease. Since IL-2 induces the activation of immunosuppressive Tregs [98], synthetic biology approaches using fusion proteins that express a variant of IL-2 that is insensitive to the IL-2 receptor found on Tregs alongside an NKG2D-binding protein aimed at selectively activating NKG2D-bearing NK cells with IL-2, while avoiding the unwanted activation of Tregs, are also being explored [99]. Another conditioning approach, by priming NK cells with tumor cells, has been shown to result in the activation of NK cells independently of IL-2 to generate NK cells able to lyse a variety of cancer targets [100].

Several investigations have addressed the concept of “optimal dose” of NK cells in adoptive transfer immunotherapy [101]. The principal confounding factor is the highly variable number of alloreactive NK cells that can be sourced from each donor. Curti et al. [102] showed that a larger infused NK cell dose, consisting of >8/100 alloreactive NK cell clones (at a dose of at least 2 × 10^5^ cells/kg) resulted in improved disease-free survival without negative bias toward patients with lower numbers of alloreactive NK cells. More investigations have since looked at optimizing NK cell doses for patients with various cancers [103].

To establish optimal NK cell preparation regimens, Besser et al. [104] compared various strategies to augment NK cell cytotoxicity within the context of allogeneic adoptive transfer. They found that combining the enhancement of NK cell activating receptor expression (NKp44, NKp46, NKp30, and NKG2D) by optimizing culture conditions, with engagement of antibody-mediated cellular cytotoxicity (ADCC) via CD16 on NK cells, and KIR-mismatch yielded the greatest NK cytotoxicity in vitro. Individually, augmenting activating receptor expression yielded the greatest enhancement in NK-mediated cytolysis of cancer cells, followed by NK cell activation via ADCC and, finally, KIR-mismatch.

However, none of these protocols is either standardized or reproducible in different patient or tumor settings. Overall, more work on identifying optimal dosing, pre-conditioning, and expansion regimens for infusions of NK cells, especially in the setting of solid tumors, is needed.

### 3.3. Umbilical Cord Blood NK Cells

Though highly variable, on average 30% of the lymphocytes in umbilical cord blood are NK cells, compared to 10% in peripheral blood [105], making cord blood a potentially useful source of NK cells for immunotherapy. Approximately half a dozen currently active clinical trials utilize cord blood-derived allogeneic NK cells (Table 3). Though cord blood contains the two main NK subsets present in peripheral blood, CD56^dim^ and CD56^bright^, differences between cord blood and peripheral blood NK cells have been described [106,107,108], such as the presence of a CD56^−^/CD16^+^ subset in cord blood that is absent from peripheral blood [109]. This subset has been shown to have both lytic function, albeit lower than that of mature CD56^+^CD16^+^ NK cells, against K562 cell targets and can be induced to express CD56 through overnight activation with IL-2 or IL-15, likely suggesting it is a close precursor to mature NK cells [110]. Additionally, studies have shown that this subset may also play a role in GvL in patients treated with cord blood-derived NK cells [111]. Cord blood offers several advantages that benefit its use in allogeneic adoptive transfer settings, among them are its relative ease of collection [112] and lower number of T cells which reduces the risk of GvHD [113]. However, a delayed immune reconstitution following umbilical cord blood transplantation (CBT) [114,115] and the incomplete maturation of cord blood NK cells [116] hint at an altered effector function for these cells. This is reflected by their lower rate of interferon (IFN)-γ production and requirement for activation with both interleukin-2 (IL) and IL-15 or IL-15 and IL-18 [117] as opposed to IL-2 alone like peripheral blood NK cells. More specifically, cord blood NK cells have been shown to have lower expression of inhibitory KIR receptors, CD158a and CD158b, and higher expression of inhibitory receptor NKG2A than that of mature peripheral-blood NK cells [116]. Additionally, there was a lower level of expression of several activating receptors and coreceptors, including DNAM-1, NKG2C, and NKp46, which is consistent with incomplete maturation of cord blood NK cells. Therefore, downregulation of KIRs, several activating receptors, and upregulation of inhibitory NKG2A, as well as lower production of cytotoxicity and proliferation ligands, such as granzyme B, perforin, and IFN-γ contribute to the limited anti-tumor effector function of cord blood NK cells. Moreover, engraftment of NK cells is earlier than T cells following CBT [118,119], suggesting that NK alloreactivity might contribute to protection from relapse.

The role of GvHD in CBT remains somewhat controversial. Rocha et al. reported that, compared to bone marrow transplantation, cord blood from matched siblings was found to have a lower incidence of GvHD compared to patients receiving bone marrow transplantation [120]. Elsewhere, Brunstein et al. found that KIR-ligand mismatch was associated with a higher incidence of grade III-IV acute GvHD in patients receiving single and double unit CBT [121]. No such direct relationship was reported by Garfall et al. [122], who examined a cohort of 80 patients who underwent double unit CBT, 35 of which were KIR ligand-mismatched and underwent either reduced intensity conditioning or fully myeloablative conditioning. They found that KIR mismatch was not associated with reduced relapse, overall survival or GvHD. A similar conclusion was reported by Tanaka et al. [123] for acute lymphocytic leukemia (ALL) and AML patients receiving single unit CBT. In light of clinical data, the use of KIR mismatch in donor selection for CBT remains unclear [124,125]. Additionally, pro-inflammatory and anti-inflammatory cytokine production by NK cells, as well as T cells and macrophages, has been shown to be reduced in cord blood when compared to peripheral blood. Furthermore, storage conditions of cord blood, which is typically stored frozen as opposed to used fresh, as in the case of peripheral blood, have been shown to enhance anti-inflammatory cytokine production, limiting the function of effector cells in cord blood [126]. These factors may contribute to lowering the immunoreactivity and incidences of GvHD in CBT, but the exact reason remains unclear.

Within the context of CBT, Escobedo-Cousin et al. [127] found that cord blood NK cells are the accessory cell type that are responsible for improving homing and clonogenicity of cord blood stem cells during CBT. A number of clinical trials are underway using cord blood-derived NK cells [109]. These cells can be isolated in a single step procedure with positive selection for CD56 due to the relatively low proportion of natural killer T (NKT) cells in cord blood. However, they are functionally immature compared to peripheral blood-derived NK cells, requiring ex vivo expansion and activation. Shah et al. [128] reported that cord blood NK cells, expanded with antigen-presenting feeder cells, in doses up to 1 × 10^8^ cells/kg were well tolerated in the setting of allogeneic adoptive transfer. Nguyen et al. [129] also studied the functional maturation of reconstituted cord blood NK cells after unrelated cord blood transplantation in patients with acute myeloid leukemia. They reported high levels of CD56^bright^ cells, overexpression of CD94/NKG2A, intracellular IFN-γ production, and downregulation of the expression of CD16, CD8, and CD57―collectively, signs of functional NK immaturity.

Cord blood NK cells have also been genetically engineered to express chimeric antigen receptors (CARs). A clinical trial (NCT03579927) is underway at MD Anderson Cancer Center evaluating the safety and efficacy of cord blood-derived NK cells expressing CD19 together with IL-15 and caspase-9-based suicide gene (iCasp9) to treat patients with B cell non-Hodgkin lymphoma undergoing high dose chemotherapy and autologous stem cell transplantation. These cells were shown to exhibit significant pre-clinical efficacy and long in vivo post-infusion persistence, with the suicide gene eliminating toxicities and potential off-target effects [130].

### 3.4. NK Cells and GvHD

The role of NK cells in GvHD induction following adoptive transfer into recipients has been the subject of much debate [131]. A number of studies have promoted the notion that NK cells, in allogeneic adoptive transfer settings, help prevent GvHD by suppressing alloreactive T cells [89,90,91]. More recent studies, however, have put forth the concept that NK cells can, in certain cases, promote GvHD, particularly with matched unrelated donor as opposed to matched sibling donor recipients [86]. One mechanism by which this is thought to occur is due to the heightened production of pro-inflammatory cytokines IFN-γ and tumor necrosis factor-α (TNF-α) from tumor-infiltrating NK cells. The significance of the dual protective-promoting role of GvHD by NK cells in allogeneic adoptive transfer settings, however, remains controversial, independently of the inclusion of a regimen of lymphodepletion of alloreactive T cells prior to NK cell infusion. Much of this is due to the fact that other compounding factors in adoptive NK cell transfers where induction of GvHD observed [86] could have contributed to the GvHD effect. In the study by Shah and colleagues [86], these include a presumed sub-optimal timing of NK cell infusion with respect to timing of HSCT engraftment, and the fact that more patients who developed GvHD showed a more rapid T cell engraftment. These patients also received grafts from unrelated donors, thus potentiating immune cell alloreactivity [131]. However, that is not to say that the role of NK cells in the induction of GvHD should be dismissed. The production of pro-inflammatory cytokines, studies have shown, can awaken T cell alloreactivity leading to elevated GvHD. This was suggested to have potentially been induced due to the administration of cytokines such as IL-2 or IL-15, given to promote NK cell proliferation and cytotoxicity [86,132]. Protocols to modify the administration of NK cells and/or cytokines have failed to show optimal administration regimens to avoid GvHD [12], however more work is needed to assess the best practices. Importantly, administration of endogenous IL-15 was shown to promote GvT effects and immune reconstitution of NK and CD8^+^ T cells in recipients of haploidentical HSCT [132]. The use of IL-15 in adoptive transfer settings has, moreover, shown to result in sustained clinical responses in a Phase I trial of an IL-15 superagonist to treat relapse following allogeneic HSCT [133]. Elsewhere, in vivo studies have identified the murine CD11b^+^ NK subset as involved in providing protection against acute GVHD [134]. Also important to consider is the role of NK cells on GvHD outside of HSCT. A number of studies have reported no onset of GvHD caused by infusions of allogeneic NK cells to treat both hematological malignancies and solid tumors, indicating that these treatments are safe and well tolerated [13,93,135,136]. Solid tumors, however, present an additional challenge: though infused allogeneic NK cells could be detectable in the blood of acute myeloid leukemia patients for up to four weeks after infusion [92], the persistence of NK cells in solid tumors is significantly lower. A study by Yang et al. [95] using random healthy-donor derived allogeneic NK cells showed lack of any severe GvHD, but recorded persistence of infused NK cells for only up to four days post-infusion.

### 3.5. NK Cell Lines

NK cell immunobiology has greatly benefited from the availability of a number of NK cell lines which have enabled the development of NK cell-based immunotherapies within the context of allogeneic adoptive transfer and without risk of GvHD. The use of NK cells lines avoids the need for leukapheresis, facilitating cell procurement, and avoiding undesirable side-effects. Among the available NK cells lines are NK-92, haNK, NKG, NKL, KHYG-1, YT, NK-YS, SNK-6, IMC-1, YTS, NKL cells as well as high affinity NK (HANK-1), an NK/T cell lymphoma cell line [137]. All of the cell lines have been derived from patients with leukemia/lymphoma and are dependent on IL-2 for the proliferation and effector functions [138]. Though all of the cell lines listed are currently being investigated, only the NK-92 cell line has progressed to clinical trials, with the NKG and KHYG-1 cell lines emerging as other promising sources of NK cells for cancer immunotherapy, though with significantly less published pre-clinical data so far. Other cell lines have so far been far less studied.

The NK-92 cell line has been the most extensively studied and is the subject of several clinical investigations. Developed by Klingemann’s group and currently licensed by NantKwest, NK-92 cells express CD56 and lack CD3, but unlike peripheral blood-derived NK cells, they do not possess CD16, and are thus unable to participate in antibody-mediated cellular cytotoxicity (ADCC) [139]. They lack some activating receptors, such as NKp44 and NKp46, and are thought to not possess inhibitory KIR receptors: KIR2DL has long been considered the only receptor that had been identified as being expressed by NK-92 [140], resulting in KIR mismatch following adoptive transfer and, as a result, a more potent lytic activity [141]. It should be noted that DNA methylation studies have suggested NK-92 possess various KIRs [142]. Additionally, due to their biological origin from a patient who suffered from acute NK cell lymphoma, NK-92 cells must be irradiated prior to infusion [143]. Even so, NK-92 cells can be genetically engineered with relative ease compared to peripheral blood-derived NK cells, which are notoriously resistant to exogenous gene uptake [40]. This allows their use as gene-modified cellular therapies, such as chimeric antigen receptors [144,145]. Pre-clinically, NK-92 cells have been the subject of multiple investigations, alone and in combination with monoclonal antibodies [146,147,148], small molecule chemotherapy drugs [149,150] or radioiodine therapy [151]. Moreover, development studies aiming at establishing optimal cytokine stimulation programs [148], optimizing media conditions [149], and elucidating the effects of chemotherapy on their cytotoxicity [150] have also been reported.

Evidence from multiple trials suggests that the clinical use of NK-92 cells is considered safe and appears well-tolerated [97,152,153,154,155,156,157,158]. Currently, there are 10 trials registered worldwide employing the NK-92 cell line. Of those, four are recruiting as of the first quarter of 2019. All of the actively recruiting trials employ NK-92 cells genetically engineered to express various receptors for cancer antigens and are designed to target glioblastoma (ErbB2-specific clone NK-92/5.28.z), non-small cell lung carcinoma (CCCR-NK-92), various refractive solid tumors (NK-92 cells modified to express CD16, termed haNK^®^ cells), and various leukemias and lymphoma (anti-CD19 NK-92 cells). Results of a first-in-man safety study using CD33-CAR-NK-92 cells engineered with CD28, 4-1BB, and CD3ζ co-stimulatory domains in patients with relapsed and refractive acute myeloid leukemia showed that doses up to 5 × 10^9^ NK-92 cells per patient could be tolerated without significant adverse effects [159]. Grade I cytokine release syndrome was reported for one of the three treated patients. Ultimately, the study did not, however, demonstrate clinical efficacy.

Because NK-92 cells do not express CD16, a related cell line has been engineered based on NK-92 precursor cells, to express the high affinity (ha) CD16 V158 FcγRIIIa receptor, as well as IL-2. Termed haNK, the cell line was shown to produce high levels of granzyme and perforin and participate in ADCC, resulting in efficient lysis of almost two dozen different tumor cell lines [160]. The Food and Drug Administration (FDA) granted haNK cells investigational new drug (IND) status in 2017. Phase I clinical studies of 300 doses of haNK cells in combination with a vaccine cocktail composed of, among others, recombinant human super agonist IL-15, nab-paclitaxel, anti-PDL1 monoclonal antibody, and anti-vascular endothelial growth factor (VEGF) monoclonal antibody, resulted in zero incidence of cytokine release syndrome. All treated patients had advanced metastatic cancers refractive to previous treatments. In the case of late-stage advanced metastatic pancreatic cancer (3rd line or greater) patients, for instance, the study recorded 90% disease control with median overall survival of 9.5 months, higher than the standard-of-care average of 8.7 months, while among papillary carcinoma patients who failed standard-of-care, 100% remain disease-free [161].

NKG is another allogeneic cell line that has shown robust pre-clinical responses against various tumors. Derived from a Chinese male patient with rapidly progressive non-Hodgkin’s lymphoma, NKG cells are CD56^+^/CD16^−^/CD3^−^, IL-2-dependent, and express activating receptors NKp30, NKp44, NKp46, NKG2D, and NKG2C [162,163]. Additionally, NKG cells secrete cytolysis related molecules such as IFN-y, granzyme B, and perforin, characteristic of activated NK cells [158]. NKG cells have also been shown to have increased cytolytic function against both MHC-I^+^ and MHC-I^-^ cancer cell lines when compared to NK-92 cells, likely due to increased NKG2D and NKp30 expression [158]. Though NKG cells are not yet used clinically, their functional characteristics when compared to NK-92 cells appear promising, and advances toward the good manufacturing practice (GMP) preparation of these cells are being pursued [164].

KHYG-1 cells [165] were derived from a female patient with aggressive NK cell leukemia [166] and are CD56^+^/CD16^−^/CD3^−^, IL-2-dependent, produce significant amounts of IFN-γ, and express activating receptors NKp44 and NKG2D [167]. These cells were recently engineered with a CAR expressing epidermal growth factor receptor variant III (EGFRvIII)-specific single chain variable fragment (scFv) coupled to CD3ζ, CD137 (4-1BB), and CD28 co-stimulatory domains [168]. When tested in vitro, these lentivirally-transduced cells displayed more pronounced killing of U87MG glioblastoma cells compared to non-transduced KHYG-1 cells.

Though there is growing interest in using NK cells lines as allogeneic effectors for adoptive immunotherapy, current limitations limit their more widespread use. These include their lack of ADCC, the need for irradiation, and their sourcing, which has limited the procurement from commercial entities for research purposes or labs at which these cells have been developed.

### 3.6. Embryonic and Induced Pluripotent Stem Cell-Derived NK Cells

Human embryonic stem cells (hESCs) and induced pluripotent stem cells (iPSCs) represent a promising source of allogenic NK cells. hESC- and iPSC-derived NK cells provide a homogenous, reproducible source of NK cells, lacking donor heterogeneity associated with NK cells derived from peripheral blood and umbilical cord blood [169,170]. Well-defined protocols for differentiation of hESCs and iPSCs into NK cells have been developed for clinical scale production of NK cells with similar functional and phenotypic characteristics to peripheral blood NK (PBNK) cells [171,172]. Knorr et al. [171] have demonstrated that hESC- and iPSC-derived NK cells express activating and inhibitory receptors similar to those of PBNK cells, including CD56, KIR, TRAIL, CD16, NKG2A, NKG2D, NKp44, and NKp46. However, iPSC-derived NK cells have been shown to express higher levels of NKG2A and lower levels of KIR than PBNK cells, which is characteristic of more immature NK cells [173]. Nonetheless, these stem cell-derived NK cells demonstrate functional characteristics of mature NK cells, including production of IFN-γ and degranulation when exposed to tumor targets [171]. Additionally, hESC- and iPSC-derived NK cells have been shown to be cytotoxic against myeloma, pancreatic, and ovarian cancer targets in vitro at levels similar to those of PBNK cells [172,174]. In vivo efficacy of hESC-derived NK cells has also been evidenced against leukemia, breast, prostate, testicular, and glioma cancer models in mice, where heightened cytolytic activity of hESC-derived NK cells was demonstrated as compared to NK cells derived from umbilical cord blood [175]. Clinical scale production and expansion of hESC- and iPSC-derived NK cells has also been described, utilizing artificial antigen presenting cells (aAPCs), without the loss of NK-cell phenotype or in vitro cytotoxicity, yielding a clinically relevant number of NK cells from significantly fewer cells than from PBNK cells [176,177,178]. Additionally, since adoptively transferred PBNK cells typically persist for about one to three weeks, hESC- and iPSC-derived NK cells offer a continuous source of NK cells that could have potential for multiple dosing from a single donor source [91]. Furthermore, by utilizing iPSC-derived NK cell lines, which lack contaminating T and B cells, HLA matching of a large number of recipients can be achieved from a relatively small number of donors, demonstrating the potential of iPSC-derived NK as an allogeneic, “off-the-shelf” source of NK cells for cancer immunotherapies [179].

Recently, Li et al. [180] demonstrated that iPSC-derived NK cells can be genetically modified to express CARs to enhance the anti-tumor immunity of these cells against a variety of tumor targets. iPSC-derived NK cells, genetically modified with a CAR containing the transmembrane domain of NKG2D, the 2B4 co-stimulatory domain, and the CD3ζ signaling domain, expressed improved degranulation, cytokine production, cytotoxicity, and increased expansion and survival. In addition, hESC- and iPSC-derived NK cells offer improved CAR transfection efficiency over PBNK cells, establishing these pluripotent cell sources as a promising approach to the development of cancer immunotherapies with genetically modified NK cells [181,182]. These factors allow hESCs and iPSCs to be used to develop a standardized, homogenous population of CAR-expressing NK cells to improve efficiency of adoptive transfer cell therapies. This has potential to spur the development of clinical therapies that are reproducible and lack the donor-associated variability present in current NK and T cell therapies.

## 4. Conclusions

Results from recent clinical trials have suggested the safety and efficacy of NK cell-based therapies in adoptive transfer settings in treating solid tumors and hematologic malignancies. Allogeneic NK cell therapies have demonstrated potential due to their relatively short in vivo persistence and depletion of alloreactive T cells. However, even with much research, the exact extent of contribution of NK cells towards GvHD is still not well understood, in large part due to limitations in sourcing allogeneic NK cells and conflicting clinical reports. Many recent clinical trials have sourced allogeneic NK cells from peripheral blood and umbilical cord blood, both in NK cell infusions and HSCT. NK cells derived from these sources are functionally mature and are relatively easy to obtain. However, infusions from these sources are not completely lacking contaminating T and B cells, providing a potential source of GvHD. In conjunction, administration of cytokines including IL-2 and IL-15 to promote NK cell proliferation in vivo can awaken alloreactive T cells, trigger the heightened production of pro-inflammatory cytokines (IFN-y and TNF-a), and promote GvHD.

Alternative sources of NK cells for clinical trials, including NK cell lines, iPSCs and hESCs have emerged as potential means of overcoming challenges associated with GvHD from donor-derived NK cell sources. These sources are entirely lacking alloreactive T cells, do not pose the same risk of GvHD as blood-derived NK cell sources, and offer improved efficiency in genetic modification. However, NK cell lines are lacking some of the inhibitory and activating receptors present in peripheral blood-derived NK cells, and their use is limited through procurement from commercial entities and labs which have developed these cell lines. iPSC- and hESC-derived NK cells are typically, functionally immature NK cells, possessing downregulated expression of KIRs and more limited cytolytic function than blood-derived NK cells.

The challenges in sourcing NK cell infusions, free of alloreactive T cells, that are functionally mature and do not mediate GvHD are evident. Although recent clinical trials have demonstrated the safety and efficacy of NK cell adoptive transfer therapies in cancer treatment, the role of NK cells in contributing to GvHD should not be overlooked. Continued success in the development of NK cell therapies is going to increasingly depend on enhancement in alternative cell sources, such as NK cell lines and stem cell-derived NK cells, and understanding NK cell functional biology.

## Figures and Tables

**Table 1:** Currently active and ongoing clinical trials with allogeneic natural killer (NK) cells within hematopoietic stem cell transplantation (HSCT).

Source of NK Cells	NK Cell Dosage	Disease	Treatment	Phase	Clinical Trial Identifier	Sponsor
Matched related or unrelated, NK-enriched donor lymphocytes	N/A	Myeloid and lymphoid malignancies	NK-cell enriched infusions with or without DUK-CPG-001 following allogeneic HSCT	II	NCT02452697	Duke University
Donor-derived allogeneic NK cells	1−2 × 10^8^ − 5 × 10^8^ NK cells/kg	Acute myelogenous leukemia	NK infusions following human leukocyte antigen (HLA)-haploidentical hematopoietic cell transplantation	II	NCT02477787	Asan Medical Center
Donor-derived allogeneic NK cells	N/A	Lymphoma, leukemia, and lymphoid malignancies	NK cell infusions following administration of GM-CSF (granulocyte-macrophage colony-stimulating factor) and rituximab	I	NCT00383994	M.D. Anderson Cancer Center
Related, HLA-haploidentical donor NK cells	N/A	Ewing sarcoma, neuroblastoma, rhabdomyosarcoma, osteosarcoma, and central nervous system (CNS) tumors	NK cell infusions following HLA-haploidentical bone marrow transplant and reduced-intensity chemo- and radiotherapy	II	NCT02100891	Monica Thakar, MD Medical College of Wisconsin
Donor-derived allogeneic NK cells	N/A	Recurrent B cell childhood acute lymphoblastic leukemia and recurrent childhood B-lymphoblastic lymphoma	Haploidentical NK cell infusions following chemotherapy and rituximab	II	NCT01700946	St. Jude Children’s Research Hospital
Donor-derived allogeneic NK cells	N/A	Lymphoma and leukemia	NK cell infusions following fludarabine and cyclophosphamide conditioning, bone marrow transplant, and post-transplant immunosuppression	I and II	NCT00789776	Fred Hutchinson Cancer Research Center
Cytokine induced memory-like NK cells	>4 × 10^6^/kg	Acute myeloid leukemia	NK cell infusion after HSCT following fludarabine, cyclophosphamide, and total body irradiation	II	NCT02782546	Washington University School of Medicine

**Table 2:** Currently active and ongoing clinical trials with allogeneic NK cell infusions (no-HSCT).

Source of NK Cells	NK Cell Dosage	Disease	Treatment	Phase	Clinical Trial Identifier	Sponsor
Haploidentical PBMC-NK cells	N/A	Acute myeloid leukemia	Decitabine and aldesleukin in combination with NK cells	I	NCT02316964	Ohio State University Medical Center
mRNA-electroporated NKG2D-chimeric antigen receptors (CAR) allogeneic NK cells (from parent or sibling donor)	N/A	Metastatic solid tumors	CAR-NK cell infusion	I	NCT03415100	The Third Affiliated Hospital of Guangzhou Medical University
CD19-CAR-NK-92 cells	N/A	CD19^+^ leukemia and lymphoma	CAR-NK-92 cell infusion	I/II	NCT02892695	PersonGen BioTherapeutics
Cytokine-induced memory-like (CIML) NK cells	Up to 10 × 10^6^ CIML-NK cells/kg	Pediatric acute myeloid leukemia (relapse after allogeneic HSCT)	Fludarabine, Ara-C, and G-CSF followed by T cell DLI 24 hours prior to infusion of CIML-NK cells	I	NCT03068819	Washington University School of Medicine
Allogeneic PBMC-NK cells from first- or second-degree relative	3 × 105, 1 × 10^6^ or 3 × 10^6^ NK cells/kg	Acute myeloid leukemia	Preparative chemotherapy prior to NK cell infusion	I/II	NCT01520558	Coronado Biosciences
HLA-mismatched PBMC-NK cells	9.9 × 10^6^ − 14.9 × 10^6^ NK cells/kg	Neuroblastoma	Cyclophosphamide and Hu3F8 MAb	I	NCT02650648	Memorial Sloan Kettering Cancer Center
Allogeneic haploidentical CD3^−^ CD56^+^ NK cells	5 × 10^7^ − 5 × 10^8^ NK cells/kg	Acute myeloid leukemia	Flu + Cyc followed by NK cell infusions with IL-2	II	NCT02763475	La Paz University Hospital
Allogeneic haploidentical CD3^−^ CD56^+^ NK cells	1.5 × 10^6^ − 1 × 10^8^ NK cells/kg	Multiple myeloma	Ex vivo expanded NK cell infusions over 30 days	I/II	NCT01040026	University Hospital Basel
Allogeneic activated NK cells (MG4101)	2 × 10^9^ − 5 × 10^9^ NK cells/kg	Acute myeloid leukemia	Flu + Cyc followed by NK cell infusions over 56 days with IL-2	II	NCT03349502	Seoul National University Hospital
Haploidentical CD3^−^ CD56^+^ PBMC-NK cells from family donors	N/A	Acute myeloid leukemia	Flu + Cyc followed by NK cell infusions with IL-2	II	NCT02229266	Dresden University of Technology
Membrane-bound interleukin 21 expanded haploidentical NK cells	N/A	Acute myeloid leukemia	Fludarabine, high-dose cytarabine, and G-CSF followed by NK cell infusions (6 doses over 14 days)	I/II	NCT02809092	Clinical Hospital of Porto Alegre
Allogeneic activated NK cells (MG4101)	1 × 10^7^ − 9 × 10^7^ NK cells/kg	Relapsed and refractory non-Hodgkin lymphoma	Fludarabine and cyclophosphamide followed by IL-2 (bi-weekly), rituximab (bi-weekly), and NK cell infusions (bi-weekly)	I/II	NCT03778619	Green Cross Labcell Corporation
haNK^TM^ cells	2 × 10^9^ – 4 × 10^9^ NK cells/kg	Solid tumors	haNK^TM^ infusions	I	NCT03027128	NantKwest
Allogeneic and autologous PBMC-NK cells	8 × 10^9^ – 10 × 10^9^ NK cells per treatment over 3 transfusions	Solid tumors	NK cell infusions, 4 total over 3 months	II	NCT02853903	Fuda Cancer Hospital, Guangzhou

Abbreviations: PBMC: peripheral blood mononuclear cells; CAR-NK: chimeric antigen receptor natural killer cell; HSCT: hematopoietic stem cell transplantation; G-CSF: granulocyte colony-stimulating factor; DLI: donor lymphocyte infusion; CIML: cytokine-induced memory-like; Hu3f8 MAb: humanized 3f8 monoclonal antibody; IL: interleukin.

**Table 3:** Currently active and ongoing clinical trials with cord blood-derived NK cells.

Source of NK Cells	NK Cell Dosage	Disease	Treatment	Phase	Clinical Trial Identifier	Sponsor
Allogeneic umbilical cord blood (banked)	N/A	Pediatric solid tumors	Cyclophosphamide and etoposide in combination with ex vivo expanded CBNK cells	I	NCT03420963	MD Anderson Cancer Center
Allogeneic umbilical cord blood (banked)	5 × 10^6^ – 1 × 10^8^ CB-NK cells/kg	Recurrent or refractory B cell non-Hodgkin’s lymphoma	HSCT, rituximab, and chemotherapy	II	NCT03019640	MD Anderson Cancer Center
CD19/iCasp9/ interleukin (IL)-2-engineered CAR-CBNK cells	N/A	B cell lymphoma s	Flu + Cyc	I/II	NCT03056339	MD Anderson Cancer Center
Allogeneic umbilical cord blood (banked)	5 × 10^6^ – 1 × 10^8^ CB-NK cells/kg	Multiple myeloma	CBNK infusion after elotuzumab, lenalidomide, melphalan, and HSCT	II	NCT01729091	MD Anderson Cancer Center
Allogeneic umbilical cord blood (banked)	N/A	Multiple myeloma	CBNK infusion after autologous HSCT, melphalan, followed by IL-2	II	NCT02955550	Celularity Inc.
Allogeneic umbilical cord blood (banked)	5 × 10^6^ CBNK cells/kg	Chronic lymphocytic leukemia	CBNK infusion after cyclophosphamide, fludarabine, melphalan, lenalidomide, rituximab, and UCB transplant	I	NCT01619761	MD Anderson Cancer Center
Allogeneic umbilical cord blood (banked)	1 × 10^7^ CBNK cells/kg	Leukemia	CBNK infusion in combination with rituximab, fludarabine, cyclophosphamide, cytarabine, filgrastim, and lenalidomide	I	NCT02280525	MD Anderson Cancer Center
Allogeneic umbilical cord blood (banked)	N/A	Leukemia, lymphoma, myeloma, and myeloproliferative diseases	CBNK cell infusion (conditional) following busulfan, fludarabine, clofarabine, ATG, rituximab, cyclophosphamide, mesna, melphalan, and UCB transplant	II	NCT02727803	MD Anderson Cancer Center

Abbreviations: CB-NK: cord blood natural killer; CAR-CBNK: chimeric antigen receptor cord blood natural killer; HSCT: hematopoietic stem cell transplantation; UCB: imbilical cord blood; ATG: anti-thymocyte globulin.
